# PLiSAGE: enhancing protein-ligand interaction prediction with multimodal surface and geometry encoding

**DOI:** 10.1093/bioinformatics/btaf608

**Published:** 2025-11-09

**Authors:** Tianci Wang, Guanyu Qiao, Guohua Wang, Yang Li

**Affiliations:** College of Computer and Control Engineering, Northeast Forestry University, Harbin, 150040, China; Faculty of Computing, Harbin Institute of Technology, Harbin, 150001, China; Faculty of Computing, Harbin Institute of Technology, Harbin, 150001, China; College of Computer and Control Engineering, Northeast Forestry University, Harbin, 150040, China

## Abstract

**Motivation:**

Accurately predicting protein-ligand interactions is fundamental to elucidating molecular recognition and has far-reaching implications in drug discovery, gene regulation, and signal transduction. Conventional methods predominantly rely on internal structural or sequence-based protein representations. While these approaches have improved predictive performance, their dependence on limited labeled data restricts the capacity to learn expressive features from structural inputs. Moreover, they often neglect the intricate geometric and chemical context encoded on protein surfaces, limiting interpretability, and hindering mechanistic insights into binding interactions.

**Result:**

Here, we present PLiSAGE, a multimodal framework that integrates 3D structural and surface geometric embeddings to enable accurate prediction of protein–ligand interactions. Central to our approach is the joint pretraining of structural and surface encoders through unsupervised contrastive learning and point cloud reconstruction. Protein surfaces are represented as segmented point cloud patches, allowing the model to capture fine-grained geometric and chemical cues. A Transformer-based encoder further captures both local and global spatial dependencies across patches. The incorporation of spatial topological information during pretraining facilitates the learning of stable, discriminative, and multi-scale protein representations, enhancing the expressive capacity of both modalities. An adaptive fusion module dynamically integrates structural and surface embeddings to yield complete and robust protein representations. PLiSAGE demonstrates superior performance over competitive baselines in binding affinity prediction and interaction classification tasks. Extensive ablation studies underscore the critical contributions of surface features and the pretraining strategy to the model’s generalization capabilities.

**Availability and implementation:**

The source code of PLiSAGE is available at: https://github.com/catly/PLiSAGE.

## 1 Introduction

Interactions between small molecules and proteins form the basis of numerous biological processes. Understanding these interactions not only facilitates the identification of potential drug targets, screening of candidate compounds, and optimization of binding affinities, but also helps elucidate molecular recognition and signal transduction mechanisms (Jia *et al.* 2023), thus advancing precision medicine and biotechnology. Traditional experimental techniques, such as X-ray crystallography (Smyth and Martin 2000) and nuclear magnetic resonance (NMR) spectroscopy can provide high-resolution structural data of protein-ligand complexes (Qiao *et al.* 2024). Despite their reliability, these approaches are often time-consuming, costly, and limited in throughput.

Recent advances in data availability, including experimentally validated protein-ligand structures, interaction patterns, and affinity data, have enabled the development of data-driven computational methods (Jia *et al.* 2025). These approaches leverage machine learning algorithms to analyze large-scale datasets, extracting patterns and features that predict protein-ligand binding affinities and interaction mechanisms with high efficiency and scalability. Compared to traditional methods, computational approaches significantly reduce costs and time while maintaining competitive accuracy (Zhang *et al.* 2025).

With the rapid advancement of biotechnology, a large volume of high-quality experimental data has been accumulated, including protein-ligand complex structures, interaction patterns (Zhang *et al.* 2024), and binding affinity data (Li *et al.* 2022). This has paved the way for data-driven computational approaches. Machine learning-based models can efficiently learn interaction patterns from such datasets, significantly reducing experimental costs and time, while maintaining strong predictive performance. Broadly, these computational approaches fall into two categories: sequence-based (Liang *et al.* 2021) and structure-based methods (Stepniewska-Dziubinska *et al.* 2018).

Sequence-based methods typically rely on one-dimensional inputs (e.g. SMILES for ligands, amino acid sequences for proteins) or two-dimensional representations (e.g. molecular graphs, contact maps). Using techniques from NLP and GNNs, these models extract features from sequences or molecular topology. For example, DeepDTA (Öztürk *et al.* 2018) uses CNNs for feature learning, DeepDTAF (Nguyen *et al.* 2021) incorporates dilated convolutions for long-range dependencies, and GraphVQA (Liang *et al.* 2021) enhances spatial representation via 2D distance and connectivity graphs. However, they struggle to capture complex 3D interactions, limiting binding pose and affinity modeling.

Structure-based methods, by contrast, utilize 3D protein-ligand structures to model spatial and physicochemical constraints explicitly (Mastropietro *et al.* 2023). Models like KDEEP (Stepniewska-Dziubinska *et al.* 2018) and Pafnucy use 3D-CNNs with voxelized inputs but are computationally heavy and lack rotational invariance. Geometry-aware GNNs such as SIGN (Zhang *et al.* 2023) and GBPNet (Aykent and Xia 2022) improve local structural learning, though many still overlook the functional role of protein surfaces.

As the surface encodes critical geometric and chemical properties essential for molecular recognition (Sverrisson *et al.* 2021), integrating surface features with global structural context can significantly improve protein-ligand interaction (PLI) predictions. Recently, geometric deep learning has emerged as a promising direction. GVP (Jing *et al.* 2021), for example, introduces operations over scalar and vector features in a global coordinate system, preserving geometric symmetries such as rotation and translation invariance. Despite these advances, challenges remain in efficiently integrating multi-scale structural features while maintaining computational efficiency, underscoring the need for further innovation in structure-based methods (Pang *et al.* 2022; Li and Liu 2023).

Recent multimodal methods seek to build richer protein representations. MFE (Xu *et al.* 2024), for example, integrates surface, structure, and sequence features via cross-attention, while MPRL (Nguyen and Hy 2024) uses specialized encoders with a learnable fusion module. Despite these advances, two fundamental limitations persist. First, their fusion strategies remain shallow, failing to capture deep synergistic effects. Second, and more critically, they neglect the power of joint self-supervised pre-training: most are purely supervised, and others pre-train encoders independently, missing the opportunity for early cross-modal alignment. This severely limits their generalization ability in low-data settings. Consequently, a unified framework that deeply integrates key structural modalities within a robust pre-training paradigm remains an open challenge.

While many structure-based methods still overlook the rich geometric and chemical information on protein surfaces, our work aims to bridge this gap, as well as the aforementioned limitations in fusion and pre-training. To this end, we introduce PLiSAGE, a deep learning framework that differs from prior work by synergistically integrating multimodal structural and surface-level features through a joint unsupervised pre-training strategy to enhance generalization and robustness. PLiSAGE employs a structure encoder based on the Geometric Vector Perceptron (GVP), which captures spatial geometry from protein graphs while maintaining equivariance. Unlike approaches that pre-train encoders separately, our parallel surface encoder, a Transformer that processes local patches, is jointly optimized with the structure encoder via contrastive learning and masked self-supervision. This enables effective representation learning from unlabeled data and strong performance in low-data regimes. For downstream tasks, an adaptive fusion module aligns and integrates these unified, pre-trained embeddings with ligand features from an MPNN to enable accurate prediction of binding affinities and ligand classification. Our primary contributions include:

- We propose an efficient and scalable multimodal feature integration framework that combines a GVP-based structural encoder with a surface encoder for capturing spatial context, enabling robust modeling of both local and global protein features with geometric invariance.
- Extensive experiments on multiple benchmark datasets demonstrate that PLiSAGE consistently outperforms existing baseline methods in binding affinity prediction and ligand classification tasks. Ablation studies further validate the rationale, effectiveness, and scalability of the proposed model.
- To the best of our knowledge, PLiSAGE is the first model to jointly encode protein 3D structures and surface features using a self-supervised pretraining strategy. By leveraging large-scale unlabeled data, it significantly enhances generalization and performance on downstream prediction tasks.

## 2 Materials and methods

**Figure 1.**
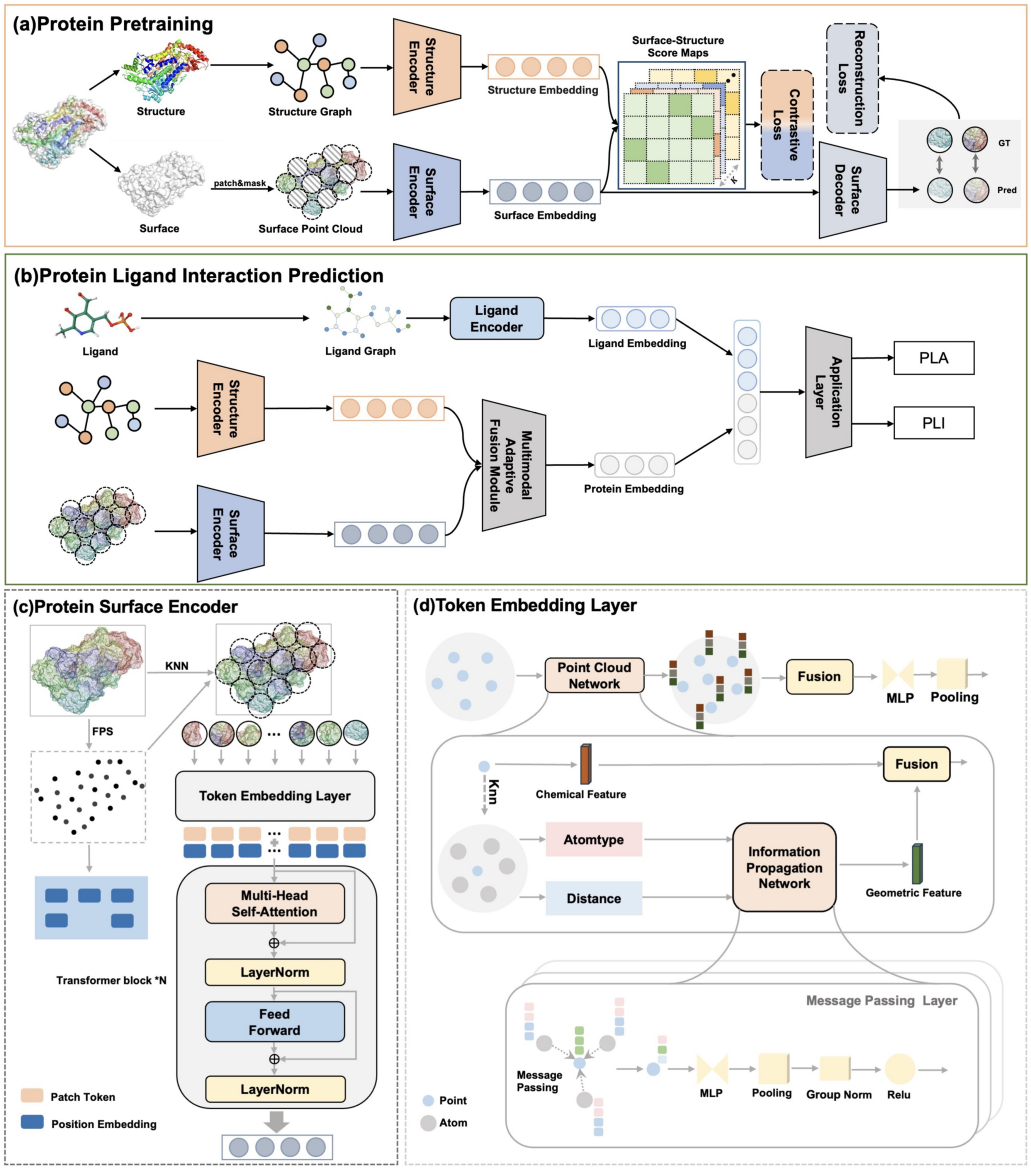
Overall architecture of the PLiSAGE framework. (a) Self-supervised Pre-training: A structure encoder (GVP) and a surface encoder (Transformer) are jointly pre-trained using two complementary objectives: a contrastive loss to align global protein representations, and a reconstruction loss to learn local surface geometry. (b) Downstream Prediction: The pre-trained protein encoders are frozen and combined with a ligand encoder (MPNN). A multimodal fusion module integrates the protein embeddings, which are then concatenated with the ligand embedding to predict Protein-Ligand Affinity (PLA) or Interaction (PLI). (c) Protein Surface Encoder: The surface point cloud is partitioned into patches, which are converted into patch tokens and processed by a Transformer architecture. (d) Token Embedding Layer: The initial embedding for each patch is generated by fusing its geometric and chemical features.

In this section, we provide a detailed description of our proposed multi-modal **P**rotein–**Li**gand interaction prediction framework, which integrates **S**urf**A**ce and **G**eometric **E**mbeddings (**PLiSAGE**). The overall architecture of the model is illustrated in Fig. 1.

### 2.1 Protein surface generation and graph construction

Recent studies have shown that the molecular surface of proteins contains critical geometric and chemical information that indicates how they interact with other molecules (Sverrisson *et al.* 2021). Sverrisson *et al.* propose dMaSIF which samples the molecular surface based on raw 3D atomic coordinates and atom types for efficient computation. Therefore, we start with a protein atomic point cloud that includes 22 atomic types and exclude atoms outside the set {C,H,O,N,S,Se}, which constitute the major atomic composition. The resulting protein point cloud consists of a set of atomic coordinates ${\left\{c_{i}\right\}}_{i=1}^{M}\in R^{M\times 3}$ and corresponding atomic types ${\left\{t_{i}\right\}}_{i=1}^{M}\in R^{M\times 6}$, where $c_{i}\in R^{1\times 3}$ denotes the centered coordinates of the *i*-th atom, and $t_{i}\in R^{1\times 6}$ represents the one-hot encoded atomic type.

To approximate the protein surface atomic point cloud ${\left\{x_{i}\right\}}_{i=1}^{N}\in R^{N\times 3}$, we use a smoothed distance function (Blinn 1982) combined with the van der Waals radii of atoms. Specifically, we associate an atomic radius $\sigma_{j}$ with each atom $c_{i}$ and define a smoothed distance function as follows:


(1) $$\mathrm{SDF}(x_{i})=-\sigma (x_{i})\cdot \;\text{log}\;\left(\sum_{k=1}^{N}\;\text{exp}\;\left(-\frac{∥x_{i}-c_{j}∥}{\sigma_{j}}\right)\right)$$

For any surface point $x_{i}$, $\sigma (x_{i})=\sum_{k=1}^{N}\;\text{exp}\;(-∥x_{i}-c_{j}∥)\sigma_{k}/\sum_{k=1}^{N}\;\text{exp}\;(-∥x_{i}-c_{j}∥)$ represents the average atomic radius within the local neighborhood of that point. We then sample points on the surface at radius *r *= 1Å by minimizing the squared loss function with respect to the level set:


(2) $$E(x_{1},\ldots ,x_{N})=\frac{1}{2}\sum_{i=1}^{N}(\mathrm{SDF}(x_{i})-r{)}^{2}$$

Subsequently, we compute initial geometric and chemical features for each surface point $x_{i}$. For chemical features, inspired by dMaSIF (Sverrisson *et al.* 2021), we select the 16 nearest atoms to $x_{i}$, and construct a feature representation $u_{i}=\{(d_{ij},\;\mathrm{type}(a_{j}))|j=1,\ldots ,16\}$ and Euclidean distances $d_{ij}=|x_{i}-a_{j}|$. For the geometric features, we utilize the smooth distance function to calculate the normal vector $n_{i}\in \;R^{1\times 3}$ of $x_{i}$. We then compute the mean and Gaussian curvatures at scales of 1Å, 2Å, 3Å, 5Å, 10Å (Cao *et al.* 2019) as the initial geometric feature $v_{i}=\{(H_{r},K_{r})|r=1Å,\;2Å,\;3Å,\;5Å,\;10Å\}$.

In addition to surface information, the internal 3D structure of a protein is modeled as a graph G=(V,E). Each node $v_{i}\in V$ corresponds to an amino acid residue, and a directed edge $e_{ji}\in E$ is drawn from residue *j* to *i* if its $C_{\alpha }$ atom is one of the k=30 nearest neighbors of the $C_{\alpha }$ atom of residue *i*.

To ensure rotational equivariance, we adopt the Geometric Vector Perceptron (GVP) GNN architecture (Jing *et al.* 2021), which requires initializing each node and edge with both scalar and vector features. Following the scheme proposed in the original GVP paper, we define the features as follows:

#### 2.1.1 Node features

For each residue *i*, denoted as $h_{v}^{\left(i\right)}=(s_{i},{\vec{v}}_{i})$, consist of a scalar part $s_{i}$ and a vector part ${\vec{v}}_{i}$. The scalar component $s_{i}\in R^{26}$ is a concatenation of a 20-dimensional one-hot vector for the amino acid type and a 6-dimensional vector containing the sine and cosine of the three backbone dihedral angles (ϕ,ψ,ω). The vector component ${\vec{v}}_{i}\in R^{1\times 3}$ is the unit vector pointing from the residue’s $C_{\alpha }$ to its $C_{\beta }$ atom (a zero vector for Glycine).

#### 2.1.2 Edge features

For a directed edge $e_{ji}$, denoted as $h_{e}^{\left(ji\right)}=(s_{ji},{\vec{v}}_{ji})$, are similarly composed. The scalar component $s_{ji}\in R^{16}$ is generated by expanding the Euclidean distance between the $C_{\alpha }$ atoms, $d(C_{\alpha ,j},C_{\alpha ,i})$, using a set of 16 Gaussian radial basis functions (RBFs). The vector component ${\vec{v}}_{ji}\in R^{1\times 3}$ is the unit vector representing the direction from $C_{\alpha ,j}$ to $C_{\alpha ,i}$, calculated as:


(3) $${\vec{v}}_{ji}=\frac{x_{C_{\alpha ,i}}-x_{C_{\alpha ,j}}}{∥x_{C_{\alpha ,i}}-x_{C_{\alpha ,j}}∥}$$

### 2.2 Protein surface spatial modeling based on point clouds

As shown in Fig. 1c, our surface encoder consists of a Token Embedding Layer and Transformer Encoder. The primary function of the Token Embedding Layer is to convert protein surface point clouds into a series of tokens, which are then fed into the Transformer Encoder (Zhang *et al.* 2023) to learn surface representations for pretraining and downstream tasks.

A straightforward approach would be to treat each point in the surface point cloud as an individual token, similar to the PointNet (Qi *et al.* 2017) method. However, due to the quadratic computational complexity of self-attention mechanisms, such point-wise tokenization results in prohibitively high computational costs, making it unsuitable for large-scale datasets. To address this, we adopt a strategy inspired by PointMAE (Pang *et al.* 2022) and PointBert (Yu *et al.* 2022), leveraging Farthest Point Sampling (FPS) to select *g* center points. For each center point, we then use the K-Nearest Neighbors (KNN) algorithm to identify *K* nearest points, partitioning the surface point cloud into *g* patches. The partitioning process can be formulated as follows:


(4) $$x_{p}=\text{FPS}(x_{p}),x_{p}\in R^{g\times 3}$$

(5) $${\left\{P_{i}\right\}}_{i=1}^{g}=\text{KNN}({\left\{x_{p}\right\}}_{i=1}^{g},{\left\{x_{j}\right\}}_{j=1}^{K}),P_{i}\in R^{K\times 3}$$

where $x_{p}$ represents the center points obtained through FPS, and $P_{i}$ denotes the patch associated with the *i*-th center point. This partitioning method significantly reduces computational complexity while preserving local geometric information, enabling more efficient and structured representation of surface point clouds.

We input each preprocessed local patch into a Token Embedding layer to generate high-dimensional representations (as shown in Fig. 1d). In this module, we process both initial geometric and chemical features. For the chemical encoding, we construct a point cloud graph using atomtypes and distances, and apply multiple message passing layers to extract higher-level representations. Geometric features are concatenated with the chemical features and passed through an MLP followed by a pooling operation to produce unified patch embeddings. To capture both local and global dependencies, we employ a Transformer encoder with positional embeddings that preserve spatial relationships among patches. The final surface-level protein representation is derived by averaging all patch embeddings $f_{\text{surface}}$.

### 2.3 Protein structural graph modeling based on GVP

Conventional graph neural networks (GNNs) primarily focus on topological connections while neglecting spatial directions and positions, which are critical for modeling protein-ligand interactions. To address this limitation, we adopt a Geometric Vector Perceptron (GVP) as our structural encoder, which inherently captures geometric symmetries such as rotation and translation invariance. The protein is represented as a graph G=(V,E), where each node corresponds to an amino acid annotated with scalar and vector features. Through the geometry-aware message passing mechanism of GVP, node features are iteratively updated to encode hierarchical structural context. In GVP-GNN, the node features are updated through the following message-passing process:


(6) $$m_{ij}={\text{GVP}}_{s}\left(\text{concat}\left(h_{v}(j),h_{e}(j\to i)\right)\right)$$

(7) $$h_{v}(i)\leftarrow \text{LayerNorm}\left(h_{v}(i)+\frac{1}{\sqrt{d}}\cdot \text{Dropout}\left(\sum_{j\to i\in \varepsilon }m_{ij}\right)\right)$$

where $m_{ij}$ represents the message passed from node *j* to node *i*, $h_{v}(i)$ represents the embedding of amino acids in the protein structure, ε is the set of all edges, and *d* is the square root of the number of incoming edges. Finally, a pooling operation is applied over the nodes in the protein structure graph to obtain the overall protein structural representation, denoted as $f_{\text{structure}}$.

### 2.4 Protein multimodal pretraining and joint optimization

In the pretraining phase, we designed an unsupervised multimodal learning framework that leverages both protein surface and structural information. The framework focuses on learning effective protein representations by combining contrastive learning with reconstruction tasks, thereby offering a robust initialization for downstream protein-ligand interaction prediction. By integrating the geometric features of protein surfaces and the topological features of protein structures, this method enhances the robustness and generalization capability of protein representations through joint optimization.

As described in Sections 2 and 2.3, we encode protein structures as graphs and protein surfaces as point clouds. The surface point cloud is divided into multiple patches. During pretraining, to fully exploit the information from both modalities and achieve the pretraining objectives—contrastive learning and surface point cloud reconstruction—we randomly mask a proportion of the patchs before feeding them into the Transformer module, enabling subsequent reconstruction predictions.

In the contrastive learning task, the unmasked patch representations are processed through the surface encoder to produce $f_{\text{surface}}$, while the global structural representation is extracted using GVP-GNN to yield $f_{\text{structure}}$. The contrastive learning objective, based on cosine similarity, is defined as:


(8) $$L_{\text{contrastive}}=-\frac{1}{N}\sum_{i=1}^{N}\;\text{log}\;\frac{\;\text{exp}(\mathrm{sim}(f_{\mathrm{surface}}^{i},f_{\mathrm{structure}}^{i})/\tau )}{\sum_{j=1}^{N}\;\text{exp}\;(\mathrm{sim}(f_{\mathrm{surface}}^{i},f_{\mathrm{structure}}^{j})/\tau )}$$

where sim(·,·) denotes cosine similarity, τ is a temperature coefficient, and *N* is the batch size. This objective maximizes modality consistency and distinctiveness, enabling the learning of cross-modal consistent protein representations.

To enhance the representation capacity of the surface encoder, we introduce a surface point cloud reconstruction task. For masked surface patches, the model generates reconstructed point clouds through the surface encoder and minimizes the Chamfer distance (Fan *et al.* 2017) between the predicted and ground truth point clouds:


(9) $$L_{\text{reconstruction}}=\frac{1}{\left|M\right|}\sum_{x\in P_{\mathrm{mask}}}\left(\underset{y\in P_{\mathrm{true}}}{\min}∥x-y{∥}^{2}+\underset{y\in P_{\mathrm{true}}}{\min}∥y-x{∥}^{2}\right)$$

Here, $P_{\text{mask}}$ represents the reconstructed point cloud, and $P_{\text{true}}$ is the ground truth point cloud.

The final pretraining loss combines the contrastive learning loss and the reconstruction loss with weighted contributions:


(10) $$L=\lambda_{1}L_{\text{contrastive}}+\lambda_{2}L_{\text{reconstruction}}$$

where $\lambda_{1}$ and $\lambda_{2}$ are hyperparameters that balance the two tasks.

Through joint pretraining of surface and structural modalities, our model effectively captures the global geometric and local chemical information of proteins. The contrastive learning task improves semantic alignment between the surface and structure modalities, while the reconstruction task further enhances the surface encoder’s ability to model local geometric patterns. Experiments demonstrate that this multimodal pretraining framework significantly outperforms single-modality methods, yielding notable performance gains on downstream tasks.

### 2.5 Protein-ligand interaction prediction

The pretraining phase of the model extracts rich surface and structural features from vast amounts of unlabeled protein data through self-supervised learning tasks. In protein-ligand interaction tasks, our model integrates specific ligand features with the structural and surface representations of proteins, using a multi-modal adaptive fusion module to effectively align the multi-modal features of the protein, resulting in a more comprehensive protein representation for predicting protein-ligand interactions. This approach captures the geometric, topological, and chemical interactions between proteins and ligands, making it especially suitable for tasks such as molecular binding affinity and binding classification predictions.

#### 2.5.1 Graph-based representation for ligand modeling

Ligands are represented as molecular graphs $G^{l}=\left(V^{l},E^{l}\right)$, where $V^{l}$ denotes the set of atoms (nodes) and $E^{l}$ represents the set of chemical bonds (edges). We utilize a Message Passing Neural Network (MPNN) to encode the molecular graph. Through an iterative message-passing process, node features are updated layer by layer, progressively incorporating chemical environment information from neighboring atoms. This method results in a high-dimensional ligand embedding $f_{\text{ligand}}$, which effectively captures both the local and global chemical properties of the molecule. The message-passing update can be formulated as:


(11) $$h_{i}^{l}(t)=\psi \left(h_{i}^{l}(t-1),\sum_{j\in N(i)}\phi \left(h_{i}^{l}(t-1),h_{j}^{l}(t-1),e_{ij}^{l}\right)\right)$$

where ϕ and ψ are the message and update functions, respectively. Finally, a global pooling operation aggregates the node features to produce the ligand embedding:


(12) $$f_{\text{ligand}}=\mathrm{Pooling}\left(\left\{h_{i}^{l}(T)|i\in V^{l}\right\}\right)$$

#### 2.5.2 Protein structural-surface adaptive fusion

In the process of fusing multimodal protein features, conventional methods, such as directly concatenating embeddings, often fail to account for inter-modal heterogeneity, which may lead to the loss of features from specific modalities. To address this issue, we propose a Transformer-based multimodal adaptive fusion module. Specifically, for the protein structure embedding $f_{\text{structure}}$ and surface embedding $f_{\text{surface}}$, we first utilize the self-attention mechanism of a Transformer Encoder to extract contextual features of the protein structure:


(13) $$\;f_{\text{structure}}^{\prime }=\text{TransformerEncoder}\left(f_{\;\text{structure}}\right)$$

Subsequently, the cross-attention mechanism of a Transformer Decoder is applied to align the surface embedding with the structure embedding:


(14) $$f_{\text{surface}}^{\prime }=\mathrm{TransformerDecoder}\left(f_{\;\text{structure}}^{\prime },f_{\text{surface}}\right)$$

Finally, the surface and structure embeddings are pooled separately, concatenated, and passed through a fully connected layer to generate a unified protein representation:


(15) $$f_{\text{protein}}=\text{FC}\left(\mathrm{Concat}\left(\mathrm{Pooling}\left(f_{\text{surface}}^{\prime }\right),\mathrm{Pooling}\left(f_{\text{structure}}^{\prime }\right)\right)\right)$$

#### 2.5.3 Protein-ligand embedding joint prediction

In the final stage of the model, the protein embedding $f_{\text{protein}}$ is concatenated with the ligand embedding $f_{\text{ligand}}$ to generate a unified complex representation:


(16) $$f_{\text{complex}}=\mathrm{Concat}\left(f_{\text{protein}},f_{\text{ligand}}\right)$$

This complex representation is then fed into a multilayer perceptron (MLP) to perform two independent tasks: affinity prediction and ligand classification.

For affinity prediction, the task aims to predict the binding affinity of a protein-ligand complex, formulated as a regression problem. The model uses the mean squared error (MSE) as the loss function, defined as:


(17) $$L_{\text{affinity}\;}=\frac{1}{N}\sum_{i=1}^{N}{\left(y_{i}-{\hat{y}}_{i}\right)}^{2}$$

where $y_{i}$ represents the ground truth binding affinity, ${\hat{y}}_{i}$ denotes the predicted value, and *N* is the total number of samples. Minimizing this loss function ensures the model improves the accuracy of affinity predictions.

For ligand classification, the goal is to determine whether a given molecule is a ligand. A Sigmoid activation function is applied to the output layer of the MLP to produce a probability ${\hat{y}}_{i}\in [0,1]$, indicating the likelihood that the molecule is a ligand. The classification task uses binary cross-entropy (BCE) as the loss function, defined as:


(18) $$L_{\text{classification}\;}=-\frac{1}{N}\sum_{i=1}^{N}\left[y_{i}\;\text{log}\;\sigma \left({\hat{y}}_{i}\right)+\left(1-y_{i}\right)\;\text{log}\;\left(1-\sigma \left({\hat{y}}_{i}\right)\right)\right]$$

where $\sigma (x)=\frac{1}{1+e^{-x}}$ is the Sigmoid function, $y_{i}\in \{0,1\}$ represents the true label, and ${\hat{y}}_{i}$ is the raw output (logits) of the model. This loss function effectively penalizes incorrect predictions, enhancing the classification performance.

## 3 Results

### 3.1 Datasets

#### 3.1.1 Pretraining dataset

We used high-quality data from the AlphaFold v2 (Varadi *et al.* 2024) protein structure database to pretrain the protein structure encoder and surface encoder. Specifically, we selected predicted protein structures from the Swiss-Prot (35, 2021) subset. This subset contains about 540k protein structures. The dataset provides detailed structural information for pretraining and helps the encoder capture geometric properties and surface interaction features of proteins.

#### 3.1.2 Affinity prediction dataset

We used the PDBbind v2020 dataset to evaluate the performance of PLiSAGE. PDBbind contains biomolecular complexes from the Protein Data Bank (PDB) with experimentally measured binding affinity data. We trained and validated PLiSAGE on the General Set and Refined Set of PDBbind v2020 (Liu *et al.* 2015). To test the generalization ability of PLiSAGE, we used the CASF-2016 (Su *et al.* 2019) dataset. CASF-2016 includes 285 high-quality protein-ligand complexes and is used to evaluate binding affinity prediction models. There is no overlap between the training, validation, and test sets.

#### 3.1.3 Interaction classification dataset

We further evaluated PLiSAGE on protein–ligand interaction classification using three benchmarks: BIOSNAP (Huang *et al.* 2021), DAVIS (Davis *et al.* 2011), and BindingDB (Wishart *et al.* 2006), which differ in scale and complexity. To ensure fair comparison, we strictly followed the preprocessing protocol of MolTrans (Huang *et al.* 2021). Since the original datasets only contain positive interaction pairs, negative samples were generated by randomly pairing proteins and ligands while excluding known positives, and then subsampled to maintain a balanced 1:1 ratio. As PLiSAGE requires 3D protein structures while the datasets only provide sequences, we employed AlphaFold2 to predict structures for all proteins. The high accuracy of AlphaFold2 predictions provides reliable structural inputs, which are crucial for enhancing classification performance.

### 3.2 Binding affinity prediction results

We evaluated PLiSAGE on the PDBBind v2020 dataset, comparing it against methods from four categories: sequence-based (e.g. GraphDTA (Nguyen *et al.* 2021), MolTrans (Huang *et al.* 2021), DrugBAN (Stepniewska-Dziubinska *et al.* 2018)), structure-based (e.g. GNINA (McNutt *et al.* 2021), SMINA (Koes *et al.* 2013), TankBind (Lu *et al.* 2022)), surface-based (e.g. dMaSIF (Sverrisson *et al.* 2021), PAE (Nguyen and Hy 2024)), and multimodal-based (e.g. Pafnucy (Stepniewska-Dziubinska *et al.* 2018), IGN (Jiang *et al.* 2021)), with the detailed results presented in Table 1. As a multimodal method integrating both structural and surface features, PLiSAGE achieves state-of-the-art performance across most evaluation metrics, including RMSE, MAE, Pearson and Spearman correlation coefficients, squared correlation coefficient ($r_{m}^{2}$), and consistency index (CI). It attains the lowest RMSE (1.345) and MAE (1.047) among all methods, and the highest $r_{m}^{2}$ (0.452), demonstrating superior ability to capture protein–ligand interactions. Its CI (0.743) is also competitive with leading models like TankBind (0.750) and SIGN (0.736). By jointly modeling global structural context and local surface interactions, PLiSAGE outperforms sequence-only models such as MolTrans (RMSE 1.599) and GraphDTA (RMSE 1.564), and shows better balance than interaction-based models; for instance, while TankBind has a slightly higher Pearson correlation (0.718), PLiSAGE outperforms it in MAE and $r_{m}^{2}$, indicating better overall generalization. Compared to surface-only approaches like dMaSIF and PAE, PLiSAGE benefits from richer spatial context, achieving lower MAE (1.047 vs. 1.136) and higher CI (0.743 vs. 0.710), highlighting the effectiveness of integrating complementary protein representations.

**Table 1.** Performance comparison on the PDBBind v2020 dataset. The best performing score in each column is highlighted in bold.

	Input Modality			Performance Metrics					
Model	Sequ.	Stru.	Sur.	RMSE↓	MAE↓	Pearson↑	Spearman↑	$r_{m}^{2}↑$	CI↑
GraphDTA	√			1.564	1.223	0.612	0.570	0.306	0.703
TransCPI	√			1.493	1.201	0.604	0.551	0.255	0.677
MolTrans	√			1.599	1.271	0.539	0.474	0.242	0.666
DrugBAN	√			1.480	1.159	0.657	0.612	0.319	0.720
DGraphDTA	√			1.493	1.201	0.604	0.551	0.312	0.693
WGNN-DTA	√			1.501	1.196	0.605	0.562	0.311	0.697
STAMP-DPI	√			1.503	1.176	0.653	0.601	0.327	0.719
ESM-2	√			1.380	1.120	0.659	0.619	0.247	0.719
IGN		√		1.404	1.116	0.662	0.638	0.385	0.730
SIGN		√		1.373	1.086	0.685	0.656	0.398	0.736
Pafnucy		√		1.435	1.144	0.635	0.587	0.348	0.707
OnionNet		√		1.403	1.103	0.648	0.602	0.381	0.717
GNINA		√		1.740	1.413	0.495	0.494	0.209	0.674
TankBind		√		1.345	1.060	**0.718**	**0.689**	0.404	0.750
SMINA		√		1.466	1.161	0.665	0.663	0.391	0.740
VAGE		√		1.485	1.209	0.594	0.547	0.246	0.690
dMaSIF			√	1.450	1.136	0.629	0.588	0.347	0.710
PAE			√	1.466	1.158	0.604	0.557	0.248	0.696
ESM-PLA	√	√		1.372	1.104	0.663	0.634	0.246	0.726
MPRL	√	√	√	1.372	1.104	0.663	0.634	0.246	0.726
MFE	√	√	√	1.445	1.134	0.673	0.664	0.272	0.728
**PLiSAGE (Ours)**		√	√	**1.345**	**1.047**	0.675	0.672	**0.422**	**0.743**

### 3.3 Interaction prediction results

In this section, we evaluate the performance of our proposed PLiSAGE model on three benchmark datasets: BIOSNAP, DAVIS, and BindingDB. We compare against several state-of-the-art baselines, including DeepConv-DTI (Lee *et al.* 2019), MolTrans, Kang *et al.* (Kang *et al.* 2022), MoDTI (Palhamkhani *et al.* 2025), and DLM-DTI (Lee *et al.* 2024). All PLiSAGE results are reported as the mean ± standard deviation over five runs with different random seeds, and the detailed metrics are summarized in Table 2.

**Table 2.** Cross-dataset performance comparison of protein-ligand interaction prediction models. The best performing score in each column is highlighted in bold.

	BIOSNAP		DAVIS		BindingDB	
Model	AUROC	AUPRC	AUROC	AUPRC	AUROC	AUPRC
DeepConv-DTI	0.883 ± 0.006	0.889 ± 0.007	0.884 ± 0.008	0.299 ± 0.011	0.908 ± 0.005	0.611 ± 0.006
MolTrans	0.895 ± 0.005	0.901 ± 0.005	0.907 ± 0.006	0.404 ± 0.009	0.914 ± 0.004	0.622 ± 0.005
Kang et al.	0.914 ± 0.004	0.900 ± 0.004	**0.942** ± **0.003**	0.517 ± 0.006	0.926 ± 0.003	0.639 ± 0.004
MoDTI	0.923 ± 0.003	**0.922** ± **0.003**	0.940 ± 0.004	0.508 ± 0.005	0.926 ± 0.003	0.663 ± 0.004
DLM-DTI	0.914 ± 0.003	0.914 ± 0.006	0.898 ± 0.006	0.406 ± 0.008	0.912 ± 0.004	0.643 ± 0.005
MPRL	0.923 ± 0.003	0.920 ± 0.004	0.941 ± 0.003	0.521 ± 0.006	0.931 ± 0.002	0.675 ± 0.004
MFE	0.919 ± 0.004	0.918 ± 0.005	0.938 ± 0.004	0.515 ± 0.007	0.929 ± 0.003	0.671 ± 0.005
**PLiSAGE**	**0.924** ± **0.003**	0.921 ± 0.002	0.911 ± 0.004	**0.529** ± **0.005**	**0.935** ± **0.002**	**0.681** ± **0.004**

Overall, PLiSAGE demonstrates highly competitive and robust performance across all three datasets. Notably, even when compared against the latest multimodal baselines, MFE and MPRL, our model shows superior or highly competitive results. On the large-scale and most challenging BindingDB benchmark, PLiSAGE achieves a state-of-the-art AUROC of 0.935 ± 0.002 and AUPRC of 0.681 ± 0.004, significantly outperforming all other methods, including MPRL (AUROC 0.931, AUPRC 0.675). On BIOSNAP, our model achieves the highest AUROC (0.924 ± 0.003), surpassing strong competitors like MoDTI and MPRL, while its AUPRC (0.921 ± 0.002) is statistically comparable to the best baseline. On DAVIS, a dataset where some methods like Kang *et al.* and MPRL show very high AUROC scores, PLiSAGE excels by achieving the best AUPRC (0.529 ± 0.005). This is particularly meaningful given the dataset’s imbalance, suggesting that PLiSAGE is more effective at correctly identifying true positive interactions. These results highlight the superior generalization capability of our structure–surface fusion and joint pre-training strategy.

**Figure 2.**
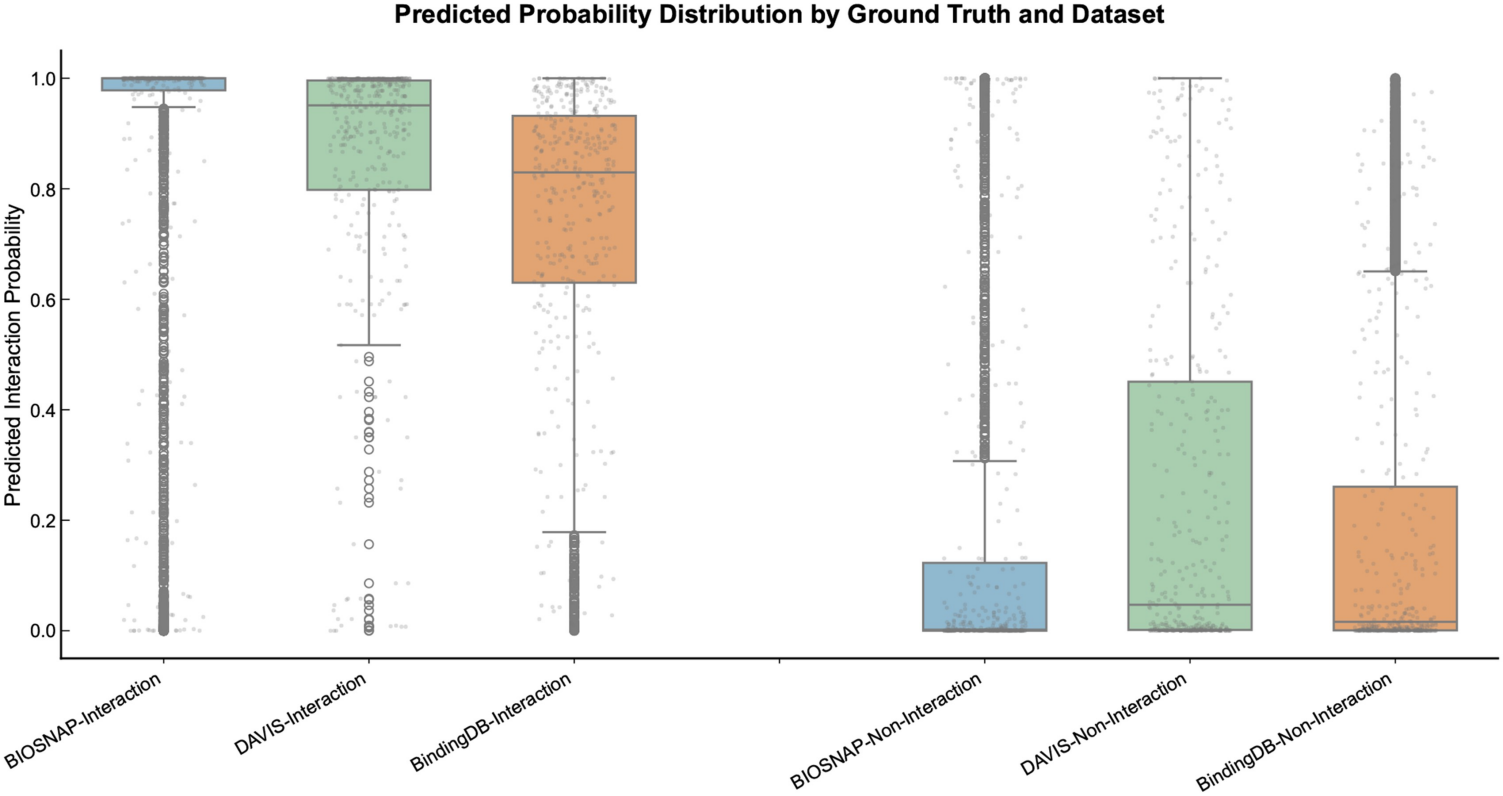
Distribution of predicted interaction probabilities on the test sets. Distribution of PLiSAGE-predicted probabilities for Interaction vs. Non-Interaction samples across three benchmarks. Grey points are individual predictions overlaid on boxplots (median, quartiles, range). The clear separation between classes highlights the model’s strong discriminative power.

To complement the summary metrics, Fig. 2 visualizes the distribution of predicted probabilities for positive (Interaction) and negative (Non-Interaction) samples. Across all three datasets, positive predictions are tightly concentrated near 1.0 and negatives near 0.0, producing a clear separation between the two classes. This visual evidence corroborates the strong AUROC and AUPRC scores, confirming that PLiSAGE learns a highly discriminative and reliable decision boundary. Collectively, while some baselines excel on specific dataset–metric combinations, PLiSAGE consistently delivers the best overall performance and robustness, particularly on large and diverse datasets, validating the effectiveness of our multimodal pre-training and fusion strategy.

### 3.4 Effectiveness analysis of feature initialization strategy

To evaluate the impact of different modules on model performance, we conducted a series of ablation experiments. We compared the performance of six model variants. The first model used only structural features (Stru.). The second model combined structural features with pretraining (Stru.+Pretrain). The third model used only surface features (Surf.). The fourth model combined surface features with pretraining (Surf.+Pretrain). The fifth model combined surface features with structural features (Surf.+Stru.). The sixth model was a complete model that combined surface features, structural features, and pretraining (Surf.+Stru.+Pretrain).

**Figure 3.**
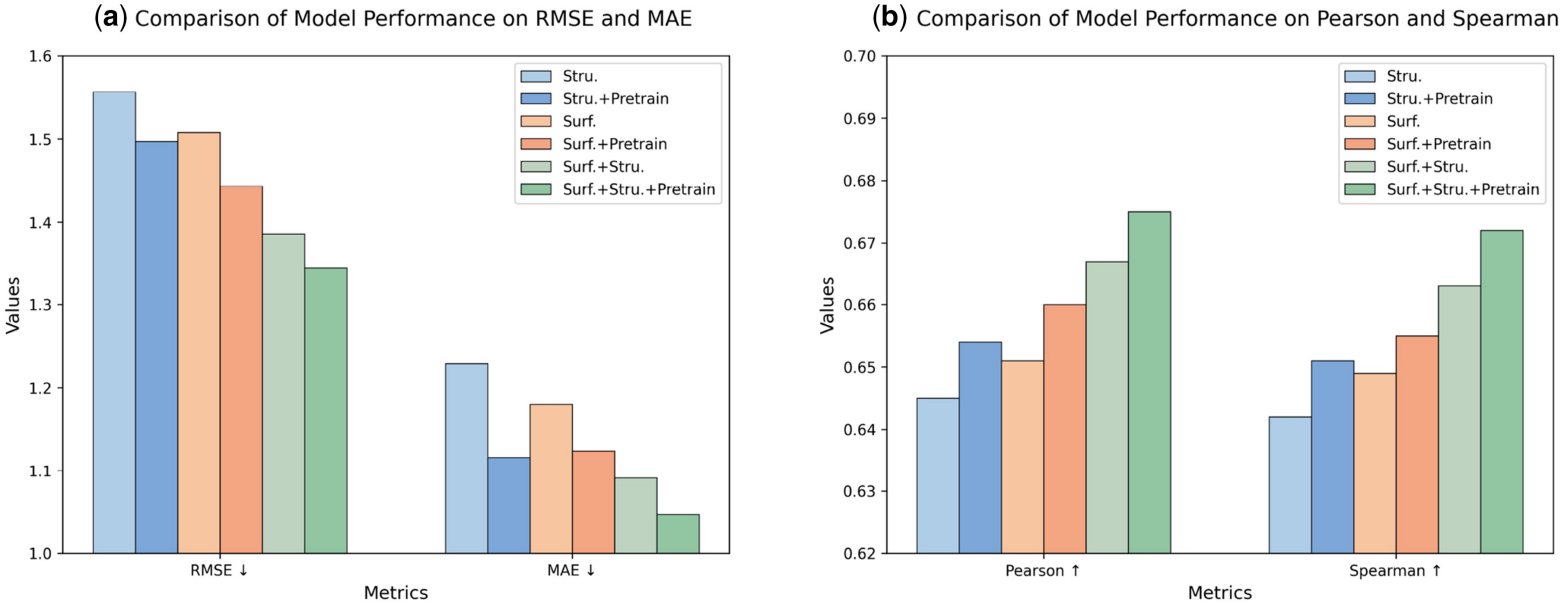
Ablation study of PLiSAGE components on the PDBbind v2020 test set. The full model (Surf.+Stru.+Pretrain) is compared with five ablated variants on four metrics, showing that surface features (Surf.), structural features (Stru.), and pre-training (Pretrain) each make complementary contributions.

We compared the performance of these variants using RMSE, MAE, Pearson correlation coefficient, and Spearman correlation coefficient. The results are shown in Fig. 3. Surface features improved model performance. They reduced error indicators such as RMSE and MAE. Pretraining strategies further improved model performance. This was true for both single-modal and bi-modal features. When surface features, structural features, and pretraining were combined, the model achieved the best performance. These features complemented each other in characterizing protein-ligand interactions. Pretraining also helped with task-related representation learning.

### 3.5 Effectiveness analysis of feature fusion strategy

To explore the impact of fusing different protein features on the protein-ligand interaction task, we evaluated three feature fusion methods. The first method used simple feature concatenation. The second method introduced a cross-attention mechanism. The third method combined self-attention and cross-attention mechanisms.

The experiment measured the performance of each model using four indicators: RMSE, MAE, Pearson correlation coefficient, and Spearman correlation coefficient. The results are shown in Table 3. The simple concatenation method performed better than other models, showing that the protein surface encoder and structure encoder learned effective protein representations.

**Table 3.** Performance of PLiSAGE fusion strategies. The best performing score in each column is highlighted in bold.

Model	RMSE ↓	MAE ↓	Pearson ↑	Spearman ↑
Concat	1.434	1.196	0.622	0.618
CrossAttn	1.402	1.159	0.645	0.637
EncDec	**1.376**	**1.114**	**0.675**	0.672^*^

When the cross-attention mechanism was added in the Transformer decoder, the model performance improved. This shows that the cross-attention mechanism captured the interaction between features and enhanced prediction ability. Using an encoder-decoder structural fusion strategy with both self-attention and cross-attention mechanisms, the model achieved the best performance.

### 3.6 Effectiveness analysis of joint optimization strategy

In the ablation experiments, Table 4 shows how different combinations of pre-training losses affect the performance of the PLiSAGE model on various datasets. Removing any pre-training loss reduces model performance on different tasks. This shows that each loss function is important in the model’s pre-training process.

**Table 4.** Ablation study of pre-training loss components.

	PDBBind^a^		BIOSNAP^b^		DAVIS^c^		BindingDB^d^	
	RMSE	MAE	AUROC	AUPRC	AUROC	AUPRC	AUROC	AUPRC
PLiSAGE (Full)	1.345	1.047	0.924	0.921	0.911	0.529	0.935	0.681
w/o Contrastive	1.433${\;}_{\left(↑6.54\%\right)}$	1.122${\;}_{\left(↑7.16\%\right)}$	0.886${\;}_{\left(↓4.11\%\right)}$	0.852${\;}_{\left(↓7.42\%\right)}$	0.851${\;}_{\left(↓6.59\%\right)}$	0.484${\;}_{\left(↓8.51\%\right)}$	0.893${\;}_{\left(↓4.49\%\right)}$	0.635${\;}_{\left(↓6.76\%\right)}$
w/o Reconstruction	1.385${\;}_{\left(↑2.97\%\right)}$	1.081${\;}_{\left(↑3.25\%\right)}$	0.913${\;}_{\left(↓1.19\%\right)}$	0.902${\;}_{\left(↓2.06\%\right)}$	0.886${\;}_{\left(↓2.74\%\right)}$	0.490${\;}_{\left(↓7.37\%\right)}$	0.925${\;}_{\left(↓1.07\%\right)}$	0.653${\;}_{\left(↓4.11\%\right)}$

Note: Values show performance metrics with percentage change (in parentheses) relative to full model configuration.

a PDBBind v2020 core set (affinity prediction task).

b BIOSNAP dataset (classification task).

c DAVIS kinase inhibition data.

d BindingDB broad-coverage interactions.

**Table 5.** Key hyperparameter settings for PLiSAGE.

Category	Parameter	Value
***Pre-training phase***		
	Optimizer	Adam
	Learning Rate	1e-4
	Weight Decay	1e-4
	LR Scheduler	ReduceLROnPlateau
	patience/factor	5/0.5
	Total Epochs	100
	Batch Size	64
	Loss Weights ($\lambda_{\text{recon}},\lambda_{\text{cont}}$)	1.0, 1.0
	Contrastive Temperature (τ)	0.05
***Fine-tuning phase (Downstream tasks)***		
	Optimizer	Adam
	Learning Rate	1e-4
	Weight Decay	5e-4
	Total Epochs	100
	Batch Size	16
***Surface encoder (PointMAE-based)***		
	Transformer Encoder Depth/Heads	10/2
	Transformer Decoder Depth/Heads	4/6
	Patch Masking Ratio	60%
	Number of Patches (num_group)	512
	Points per Patch (group_size)	32
***Structure encoder (GVP-GNN)***		
	GVP Layers	3
	Node Hidden Dims (scalar/vector)	128/32
	Edge Hidden Dims (scalar/vector)	32/1
	Dropout Rate	0.3
***Ligand encoder (MPNN)***		
	MPNN Layers	3
	Hidden Channels	64
	Dropout Rate	0.3
***Fusion module (Transformer)***		
	Model Dimension (d_model)	128
	Number of Heads (nhead)	8
	Encoder/Decoder Layers	1/1
	Dropout Rate	0.3

Removing the contrast loss significantly lowers the model’s performance in protein-ligand interaction tasks, especially in classification. This shows that the contrast loss helps inject protein attribute information and improves representation learning. Removing the reconstruction loss also affects the model, but the impact is less than that of removing the contrast loss. The reconstruction loss helps preserve the model’s original representation ability and improves its ability to capture structural information. These results confirm that each pre-training loss contributes to the model by adding protein attribute information in different ways.

**Figure 4.**
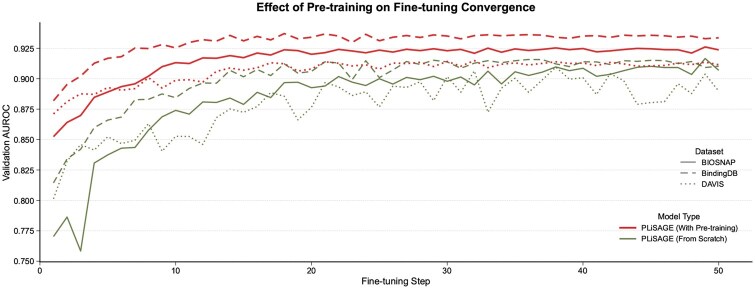
Effect of pre-training on fine-tuning convergence. Validation AUROC curves for PLiSAGE models with pre-trained initialization (red) versus random initialization (green) across three datasets. Pre-trained models show better starting performance, faster convergence, higher plateau, and greater stability, underscoring the effectiveness of our pre-training strategy.

In addition to the performance impact of individual loss components shown in Table 4, we further investigated the overall effect of our pre-training strategy on the training dynamics, as visualized in Fig. 4. The learning curves clearly illustrate the profound benefits of pre-training. For all three datasets, the pre-trained PLiSAGE models (red curves) not only converge to a significantly higher final AUROC but also do so much more rapidly and with greater stability than their counterparts trained from scratch (green curves). The models trained from scratch exhibit considerable volatility and require more training steps to reach their suboptimal performance ceiling. This visually confirms that our joint optimization strategy produces highly effective representations that accelerate and improve downstream adaptation.

### 3.7 Case study for attention guided binding site localization

To interpret and validate the learning of our PLiSAGE model, we performed a case study by visualizing its attention scores on a well-characterized protein-ligand complex: the main protease (M${\;}^{\text{pro}}$) of SARS-CoV in complex with a designed inhibitor (Yang *et al.* 2005). This system (e.g. PDB ID: 1WOF) serves as an excellent benchmark, as its key interacting residues are well-documented. The complex was rendered using PyMOL (DeLano *et al.* 2002).

**Figure 5.**
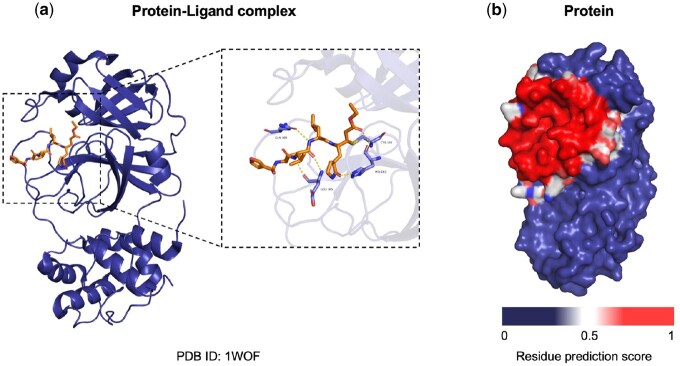
Visualization of PLiSAGE’s attention mechanism on SARS-CoV M${\;}^{\text{pro}}$. The case study is on a complex of SARS-CoV main protease (M${\;}^{\text{pro}}$) with a designed inhibitor (e.g. PDB ID: 1WOF) from Yang *et al.* (2005). (a) A detailed view of the binding pocket. The model correctly highlights key active site residues like CYS-145, HIS-163, GLU-166, and GLN-189, which are critical for ligand interaction. (b) The overall protein surface, where the model’s attention heatmap (red = high attention) accurately localizes the binding site.

As shown in Fig. 5, PLiSAGE’s attention mechanism accurately localizes the enzyme’s active site. Figure 5(b) shows that the high-attention regions (red heatmap) on the protein surface precisely overlap with the known substrate-binding pocket. Figure 5(a) provides a detailed view of this pocket, where the model correctly highlights residues crucial for inhibitor binding. Specifically, our model assigns high attention scores to catalytic residues such as CYS-145 and key binding site residues including HIS-163, GLU-166, and GLN-189. These residues are known to form a network of essential hydrogen bonds and other interactions with the ligand, as confirmed by the original structural study (Yang *et al.* 2005).

The visualization of attention highlights the ability of the PLiSAGE model to learn and pinpoint physically meaningful interaction patterns. This demonstrates that our model does not merely fit the data but captures the underlying principles of molecular recognition, providing strong support for its use in locating key functional residues.

### 3.8 Hyperparameter settings

This section lists the key hyperparameters used for the pre-training and fine-tuning of PLiSAGE, extracted from our experiment configuration files. As shown in Table 5, the hyperparameters cover critical settings such as learning rate, batch size, and fusion layer dropout rate, which collectively ensure the stability and effectiveness of the model training process.

## 4 Conclusion

In this paper, we introduced PLiSAGE, a multimodal deep learning framework designed to predict protein–ligand interactions by integrating structural and surface-level information. By leveraging unsupervised contrastive learning and point cloud reconstruction for pretraining, PLiSAGE is capable of learning robust and discriminative protein representations even with limited annotated data. The framework utilizes geometric vector perceptrons (GVP) to capture invariant features from the 3D protein structure and incorporates a modified PointMAE architecture alongside Transformers to effectively model critical geometric and chemical characteristics of the protein surface. Empirical results on benchmark datasets such as PDBBind v2020 and BIOSNAP demonstrate that PLiSAGE consistently outperforms existing state-of-the-art methods in both binding affinity prediction and interaction classification tasks, validating the efficacy and innovation of the proposed approach. Notably, the integration of surface-level insights significantly enhances predictive performance, uncovering molecular interactions often overlooked by conventional structural models.

Looking ahead, the design of PLiSAGE opens several promising research avenues. The framework could be extended to model multimeric protein interactions by adapting the surface encoder to focus on subunit interfaces, though the key challenge is to distinguish specific binding contacts from non-specific ones. Integrating PLiSAGE with molecular dynamics (MD) simulations by training on conformational ensembles could capture protein flexibility, but this poses significant challenges in computational cost and feature aggregation from numerous snapshots. Furthermore, the pre-training strategy itself could be enhanced by incorporating new modalities like evolutionary data, or by optimizing objectives for more complex tasks such as predicting binding kinetics ($k_{\text{on}}/k_{\text{off}}$ rates), which remains a critical research frontier.

## Data Availability

All data used in this study are publicly available. **Pre-training Data:** The protein structures used for the self-supervised pre-training phase were sourced from the AlphaFold Protein Structure Database (AFDB), which is available at https://alphafold.ebi.ac.uk/ and licensed under CC-BY 4.0. Specifically, we utilized both the proteome-wide predictions and the Swiss-Prot predictions. **Downstream Task Data:** The PDBbind v2020 dataset for the affinity prediction task was obtained from its official website (http://www.pdbbind.org.cn/). The BIOSNAP, DAVIS, and BindingDB datasets for the interaction classification tasks were sourced from the repository of MolTrans, available at https://github.com/kexinhuang12345/MolTrans/tree/master/dataset. **Code:** The complete source code for our PLiSAGE model, including scripts for data preprocessing and training, is publicly available on GitHub at https://github.com/catly/PLiSAGE.
